# Anesthetic efficacy of propofol combined butorphanol in laparoscopic surgery for ectopic pregnancy: A protocol of systematic review and meta-analysis

**DOI:** 10.1097/MD.0000000000020289

**Published:** 2020-05-15

**Authors:** Wang-yan Chang, Hai-ying Li

**Affiliations:** a Department of Gynecology and Obstetrics; b Department of Anesthesiology, Yan’an People's Hospital, Yan’an, China.

**Keywords:** anesthetic effect, butorphanol, ectopic pregnancy, laparoscopic surgery, propofol, safety

## Abstract

**Background:**

Recent studies have suggested that propofol combined butorphanol (PB) has anesthetic effect in laparoscopic surgery (LS) for ectopic pregnancy (EP). But investigations of its potential effects are inconsistent. We will explore the current literature examining PB in LS for EP.

**Methods:**

We will perform a comprehensive search from MEDLINE, Embase, Cochrane Library, PsycINFO, Global Health, Web of Science, Allied and Complementary Medicine Database, and China National Knowledge Infrastructure from inception to the present. Other literatures, such as conference abstracts, references to the relevant reviews will also be checked. Two authors will check the titles, abstracts, and full texts independently. They will also independently carry out data collection and study quality assessment. We will conduct statistical analysis using RevMan 5.3 software.

**Results:**

This study will provide accurate results on the anesthetic effect and safety of PB in LS for EP.

**Conclusion:**

This study will establish high-quality evidence of the anesthetic effect and safety of PB in LS for EP to facilitate the clinical practice and guideline development.

**Study registration number:**

INPLASY202040044.

## 1 Introduction

Ectopic pregnancy (EP) is a known complication of pregnancy that often causes high maternal mortality and morbidity, and fetal loss.^[1,2,3]^ Patients who experience such condition often manifest as pain, vaginal bleeding, or more vague complaints.^[4,5,6]^ Published studies have reported that EP accounts for about 2% of pregnancies, and its incidence is about 1.5% to 2%.^[7,8,9,10]^ Thus, early diagnosis and effective treatment of EP is very important.^[11,12]^

Laparoscopic surgery (LS) is widely used for the treatment of EP.^[13,14,15,16]^ During the process of LS, anesthetic medication is very important. Previous studies have found that propofol combined butorphanol (PB) can effectively manage EP patients under LS.^[17,18,19,20,21,22,23]^ However, no systematic review has been identified to assess the anesthetic effect and safety of PB in LS for EP.

## 2 Methods

### 2.1 Study registration

This study has been registered prospectively on INPLASY202040044. We report this study according to the Preferred Reporting Items for Systematic Reviews and Meta-Analyses Protocol 2015 statement.^[24]^

### 2.2 Criteria for including studies

#### 2.2.1 Types of studies

We will review randomized controlled trials (RCTs) of anesthetic effect and safety of PB in LS for EP for inclusion. Animal studies, descriptive studies, case studies, noncontrolled trials, and quasi-RCTs will be excluded in this study.

#### 2.2.2 Types of interventions

In the experimental group, all patients received PB intervention.

In the control group, all participants underwent any interventions, except PB.

#### 2.2.3 Types of patients

All female participants who were diagnosed as EP under LS and received PB will be included in this study with no restrictions of country, race, and age.

#### 2.2.4 Types of outcome measurements

The primary outcome includes pain intensity, as measured by any pain scales, such as numerical rating scales.

The secondary outcomes consist of analgesic consumption; concurrent medication; laboratory parameters; quality of life, as checked by any relevant tools, such as 36-Item Short Form Survey; and any adverse events.

### 2.3 Search strategy

We will comprehensively search following databases from inception to the present: MEDLINE, Embase, Cochrane Library, PsycINFO, Global Health, Web of Science, Allied and Complementary Medicine Database, and China National Knowledge Infrastructure from inception to the present. All electronic databases will be searched without limitations of language and publication status. The search terms include ectopic pregnancy, extrauterine pregnancy, surgery, operation, laparoscopic surgery, pain intensity, anesthetic effect, propofol, anesthesia S/I-60, anesthesia S/I-40, anesthesia S/I-40A, butorphanol, and stadol. We will list a search strategy for MEDLINE in Table 1. Similar search strategies will be modified for other different databases.

**Table 1:**
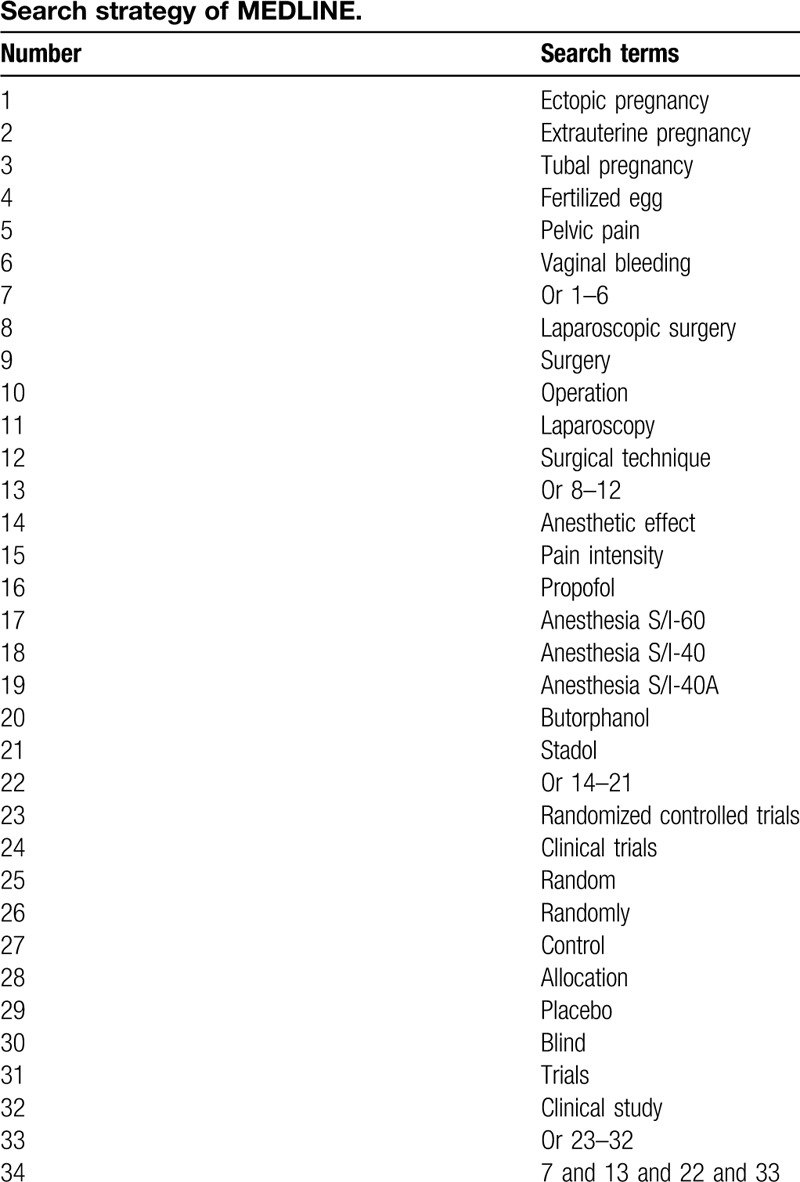
Search strategy of MEDLINE.

To avoid missing any potential studies, we will search grey literatures, such as conference abstracts, and references of relevant reviews.

### 2.4 Data collection and analysis

#### 2.4.1 Study selection

Two authors will screen the tiles and abstracts of all identified records independently and respectively. All irrelevant records will be removed. Then, we will obtain full texts of all remaining studies fulfilling the eligible criteria, and will check all of them for inclusion. Any different opinions in the checking process will be adjudicated by a 3rd author. We will present the whole process of study selection in a flowchart, and any excluded studies will be recorded with specific reasons.

#### 2.4.2 Data extraction

Two authors will independently extract data based on the standard previously defined data extraction sheet to ensure the integrity of the process. Any different views between 2 authors will be solved by a 3rd author through discussion. The following information will be extracted: study title, 1st author, year of publication, country, inclusion and exclusion criteria, diagnostic criteria, race, age, sample size, study setting, study methods, treatment details, outcome measurements, safety, and any other relevant information. If any data are missing or unclear, we will contact original authors to obtain or clarify it.

#### 2.4.3 Risk of bias assessment

We will evaluate the risk of bias from the entered studies using Cochrane risk of bias tool for RCTs, and all 7 relevant fields of bias will be checked. Each one will be further identified as low, unclear or high risk of bias. Two authors will independently assess the risk of bias, and any discrepancies between 2 authors will be examined by a 3rd author through discussion to make a decision.

### 2.5 Data synthesis

We will apply RevMan 5.3 software to perform statistical analysis. All dichotomous data will be calculated using risk ratio and 95% confidence intervals, while all continuous data will be expressed using mean difference or standardized mean difference and 95% confidence intervals. We will *I*^2^ statistics to identify potential heterogeneity among included studies and will be explained as follows: *I*^2^ ≤ 50% means low heterogeneity, and a fixed-effects model will be imposed; while *I*^2^ > 50% exerts obvious heterogeneity, and a random-effects model will be used. If low heterogeneity will be found among the eligible studies, we will perform meta-analysis on the same interventions, controls, and outcomes. If obvious heterogeneity will be identified, we will carry out subgroup analysis to check if there are some possible reasons for obvious heterogeneity. In addition, if it is possible, we will undertake a narrative description of the outcome results using detailed written commentary base on the different target patients, treatment details, controls, and outcome measurements.

#### 2.5.1 Subgroup analysis

Subgroup analysis will be undertaken based on the different study and patient characteristics, study quality, treatments, controls, and outcomes.

#### 2.5.2 Sensitivity analysis

Sensitivity analysis will be carried out to check the stability of outcome results by removing low-quality studies.

#### 2.5.3 Reporting bias

Reporting bias will be identified through funnel plot and Egger regression test when sufficient studies are entered in this study, normally at least 10 RCTs.^[25,26]^

## 3 Discussion

Numerous studies have reported the anesthetic effect and safety of PB in LS for EP.^[17,18,19,20,21,22,23]^ However, there are still contrary results of these studies, and there is not systematic review reporting the anesthetic effect and safety of PB in LS for EP. Therefore, this study will systematically and comprehensively investigate the anesthetic effect and safety of PB in LS for EP. The findings of this study will present beneficial evidence for the clinical practice and will provide helpful clue for the future research.

### 3.1 Ethics and dissemination

Ethical documents are not required for this study, because it will not inquire personal patient data. We will publish this study in a peer-reviewed journal.

## Author contributions

**Conceptualization:** Wang-yan Chang, Hai-ying Li.

**Data curation:** Wang-yan Chang, Hai-ying Li.

**Formal analysis:** Wang-yan Chang, Hai-ying Li.

**Funding acquisition:** Hai-ying Li.

**Investigation:** Hai-ying Li.

**Methodology:** Wang-yan Chang.

**Project administration:** Hai-ying Li.

**Resources:** Wang-yan Chang.

**Software:** Wang-yan Chang.

**Supervision:** Hai-ying Li.

**Validation:** Wang-yan Chang, Hai-ying Li.

**Visualization:** Wang-yan Chang, Hai-ying Li.

**Writing – original draft:** Wang-yan Chang, Hai-ying Li.

**Writing – review & editing:** Wang-yan Chang, Hai-ying Li.
